# Commentary: The antiviral and antimicrobial activities of licorice, a widely-used Chinese herb

**DOI:** 10.3389/fmicb.2016.00531

**Published:** 2016-04-18

**Authors:** Salvatore Chirumbolo

**Affiliations:** Department of Medicine-Unit of Geriatry, University Laboratories for Medical Research, University of VeronaVerona, Italy

**Keywords:** licorice, immune system, dendritic cells, flavonoids, terpenoids

A recent paper by Wang et al. about the anti-microbial and anti-viral properties of natural products should raise the concern how much organic acids, polyphenols, and terpenoids contained in herbal raw extracts, are powerful against microbial or viral pathogens-mediate infections (Wang et al., 2015). Usually, in the Traditional Chinese Medicine, licorice is known as *Gan-Cao*, coming from extracts of dried roots of *Glycyrrhiza uralensis, Glycyrrhiza glabra, and Glycyrrhiza inflata*, of which chemical analyses were performed (Zhang and Ye, 2009; Qiao et al., 2015). This paper gives me the opportunity to address some hot issue.

According to these authors, 18-β-glycyrrhetinic acid and glycyrrhizin possess an anti-viral potential, assessing previous evidence about the antiviral properties of *Glycyrrhiza* species (Fiore et al., 2008; Sabouri Ghannad et al., 2014). However, likewise any plant raw extract, the biological activity of a complex mixture of organic molecules is a non-predictable result of both bimodal and pleiotropic potential possessed by the many phenolic or terpenoid derivatives present in the extract (Wink, 2015). Although the authors focused on only six compounds among 20 triterpenoids and nearly 300 flavonoids to show evidence about their anti-viral and anti-microbial activity, questions remain if licorice is really effective either as food or herbal medicine or in the form of isolated purified molecules as active principles. This is the current concern about most of existing natural products.

Molecular interactions between 18-β-glycyrrhetinic acid and glycyrrhizin modulates the inhibition of several drug-metabolizing enzymes and efflux transporters (Feng et al., 2015), adsorption of licorice active triterpenes depends on sugars and bioavailability is better for licorice than purified glycyrrhizin (Hou et al., 2005a,b). Flavonoids in licorice act as most of flavonoids present in other plant-derived extracts, i.e., inhibiting or promoting apoptosis, switching off or modulating survival signaling pathways, increasing cytotoxicity in cancer cells. Liquiritin, isoliquiritin, and isoliquirigenin significantly increase cytotoxicity in non-small lung cancer cell line A459, up-regulate p53 and p21, decrease the expression of PCNA, PARP, Bcl-2, p-Akt, p-GSK-3β, pro-caspases (8 and 9), and downregulate the apoptotic pathways, hence encompassing a wide space of target activity (Zhou and Ho, 2014). Furthermore, the “Janus-like” behavior of many compounds contained in *Glycyrrhiza* extracts contributes in hampering a full comprehension of their beneficial effects. Licocalchone A downregulates the inflammation-induced P450 1B1 while isoliquiritigenin exerts an opposite effect, then differentially influencing the role of estrogens in chronic inflammation and in carcinogenesis (Dunlap et al., 2015). The same glycyrrhizic acid has been associated to a panoply of different beneficial effects (Ming and Yin, 2013). Wang and coworkers showed in theit thorough paper, how many anti-inflammatory properties against microbes and viruses have been attributed to licorice, though clinical trials are quite scanty.

The authors did not address either clinical trial about licorice activity against microbes.

*Glycyrrhiza-*derived purified substances, such as glabridin, are recently addressed as promising tools to prevent bacterial infections and against parasites (Cheema et al., 2014; Singh et al., 2015). Glabridin is an isoflavane, such as equol (4,7-isoflavandiol), which is a metabolite from daidzein, a well-known soy-derived isoflavonoid likewise genistein. These components are potent phytoestrogens, e.g., liquiritigenin and are able to behave as like as estradiol with estrogen receptors ERα and ERβ (Gong et al., 2014). Wang et al. described the role exerted by some triterpenes and flavonoids, such as chalcones, in inducing an anti-microbial or anti-viral, more generally an anti-inflammatory condition to prevent the onset of infections. Yet, these molecules may promote the immune response just acting as natural phytoestrogens (Kovats, 2015). A suggestion about the possible anti-microbial role of licorice phytoestrogens may come from the role exerted by estrogens and estrogen receptors on dendritic cells (Kovats, 2012). The hormone 17β-estradiol, to which plant phytoestrogens are functionally similar, regulates GM-CSF- or Flt3 ligand-driven dendritic cell (DC) development via the signaling of ERα in myeloid progenitors (Seillet et al., 2013), and moreover it regulates DCs function in immune reactions (Douin-Echinard et al., 2008). Actually, both 18-β-glycyrrhetinic acid and its alpha isomer, appears to promote DCs maturation and activity (Bordbar et al., 2014), so playing a fundamental role in immune response to microbes (Wang et al., 2015) but controversial results would suggest that the role of these compounds as phytoestrogens is not finely regulated as endogenous hormones and deserves further investigation (Kim et al., 2013). For example, despite its phytoestrogen-like property, glabridin seems to inhibit rather than promote DCs maturation and the expression of CD40, CD80, CD86, MHC-I, and MHC-II (Kim et al., 2010), though it is considered a phyto-SERM (Simmler et al., 2013).

Moreover, the role exerted by licorice in blood pressure may influence immunity and DCs functionality, as the equilibrium between the inflammatory response induced by T cell and T cell suppressor responses is critical for the regulation of blood pressure levels (Rodríguez-Iturbe et al., 2014; Ottenbacher and Blehm, 2015), although this speculative hypothesis merits to be further highlighted. Yet, despite the great deal of evidence reported by Wang et al. the presumptive anti-microbial potential of licorice overshadows even bewildering and unsuspected adverse effects (Yasue et al., 2007; van Beers et al., 2011; Yoshino et al., 2014).

This evidence supports the idea that the introduction of a whatsoever can be considered as a beneficial phytochemical from raw plants in the highly complex network of signaling interactions between cells and organs, even considering the different individual ability coming from genetics and phenotypic polymorphism or from epigenetics and lifestyle, cannot allow physicians, as well as researchers, to foresee the “real” effect of that substance in the whole organism. An interesting hypothesis is that phytochemicals, as toxic substances, produced by plants to protect themselves are beneficial only in a restricted range of doses. In this perspective, there is interesting evidence about the role of low doses of phytochemicals, particularly flavonoids, on stress response and immune reactions *in vitro*, yet surely clinical evidence is needed.

## Author contributions

The author confirms being the sole contributor of this work and approved it for publication.

### Conflict of interest statement

The author declares that the research was conducted in the absence of any commercial or financial relationships that could be construed as a potential conflict of interest.
